# Medicinal Plants Extracts with Antiangiogenic Activity: Where Is the Link?

**DOI:** 10.34172/apb.2020.045

**Published:** 2020-05-11

**Authors:** Zohreh Hoseinkhani, Fathemeh Norooznezhad, Mohsen Rastegari-Pouyani, Kamran Mansouri

**Affiliations:** ^1^Medical Biology Research Center Medical Sciences, Health Technology Institute, Kermanshah, Iran.; ^2^Student Research Committee, Department of Immunology, School of Medicine, Shahid Beheshti University of Medical Sciences, Tehran, Iran.

**Keywords:** Angiogenesis, Plant extract, Natural compounds, Chemotherapy

## Abstract

Angiogenesis is a strictly controlled process defined as the formation of new blood vessels essential for certain physiologic and pathologic conditions where the latter includes tumor growth, development, and metastasis. Thus, inhibiting angiogenesis along with other anticancer strategies such as chemotherapy seems to be invaluable for reaching an optimal outcome in cancer patients. It has been shown that some natural plant-derived compounds are capable of preventing the formation of these new blood vessels in the tumor and also inhibit the proliferation and growth of the cancer cells. In this review, we intend to introduce plants with anti-angiogenic properties and discuss their related features.

## Introduction

Man has long worked to fight the difficulties he encounters through his life and has tried various methods to control diseases. These efforts include investigating medicinal herbs which have long been used to cure diseases and still continue to keep their advantages despite the technological and scientific advances and the ever-growing use of chemical substances in modern medicine.^1,2^ Regarding the medicinal value of these herbs, their abundance, as well as the fact that most of them are unknown; the need for more research on these compounds is well tangible.^3^ Angiogenesis is the creation of new blood vessels from preformed ones in order to provide cellular needs. Angiogenesis plays an unquestionable role in some (certain) physiologic processes such as wound healing, diabetes, as well as pathologic conditions of tumor growth and metastasis.^4,5^ According to the significance of this cascade in cancers, researchers seek to find derivative compounds from herbal sources to inhibit angiogenesis. Due to fewer side-effects of herbal compounds in the treatment of angiogenesis-related disorders, investigations on plants in order to identify and discover such compounds could be a quite promising therapeutic approach. Use of herbs as treatment for cancer has a long history; so that herbs have been the primary source of traditional medications for treatment of various diseases. Although the real compounds derived from plants might not usually be utilized as medications, plants are still considered important sources for development of novel therapeutic factors for researchers. Herbal-rich food regimen not only provides the body with necessary vitamins and minerals, but also there are over 25 000 chemical compounds in different herbs; most of which hold biological effects and characteristics.^6,7^ The molecules in herbal compounds are able to bind the therapeutic agents to carrier molecules. These plant-derived tumor-targeting complexes shape the hope for producing natural drugs more effectively and highly toxic to tumors, but not normal tissues. After the identification of new proteins with important regulatory effects on the tumors’ cell cycle advancement, researchers have proven that the molecules derived from plants and other organisms could be among important inhibitors sources of synthesis or function of these key proteins. Therefore, these herbs have the potential to bring about developing novel anti-cancer agents and medications.^7-9^

Angiogenesis is the formation of new capillaries from primary blood vessels and is involved in some pathologic processes such as tumor growth and metastasis. It is also play role in certain physiologic processes like organ growth and development as well as wound healing. Angiogenesis is a necessary procedure in natural physiology of the body and if the equilibrium between its inducers and inhibitors is disturbed, opportunity will be provided for some diseases to arise.^10-13^ Angiogenesis in tumors is intervened by targeting several molecules including nitric oxide (NO) which is a critical intermediate in angiogenesis that enhances endothelial cell survival, proliferation, and migration; so, it is basically considered a pro-angiogenic factor.^14,15^ On the other hand, some of the growth factors such as vascular endothelial growth factor (VEGF) are highly specific for endothelial cells while some others such as matrix metalloproteinases (MMPs), and basic fibroblast growth factor (bFGF) have a broader spectrum of target cells. Activating factors can be secreted by tumor cells, the surrounding tissues, fibroblasts, or by macrophages which enter the tumor microenvironment. Currently, the use of VEGF pathway inhibitors in angiogenesis is considered as an anti-cancer treatment strategy with clinical credit.^16-23^

### The role of herbs in the inhibition of angiogenesis

Plants contain several active chemical compounds simultaneously and unlike chemical drugs, they can have synergistic effects and therefore, influence different aspects of disease pathology at the same time. In another words, plant extracts rich in biologically active compounds can slow down the growth of cancer cells and induce apoptosis in them at the same time which leads to tumor eradication by hindering angiogenesis and therefore, metastasis. Interactions of the active ingredients in plants extracts with tumors can give this opportunity to the immune system to identify and respond to the tumor cell.^24-26^

Nowadays, the importance of food of plant origins in preventing various diseases such as cancer, which depends on angiogenesis for growth, has been well proven. However, further studies are still required in order to study and discover more therapeutic plants with anti-angiogenic effects. Table 1 illustrates a list of plants and their major derivatives which inhibit the function of cyclo-oxygenase (COX) enzyme (one of the most important active enzymes in the angiogenesis process pathway).^12,27,28^ Table 2 shows some of the discovered plants and their derivatives which inhibit VEGF.^27^ Studies have shown, fewer side-effects of herbal compounds could be imagined compared to the chemicals and anti-cancer agents. However, there are diverse unwanted effects regarding the application of crude plant extract in in vivo condition. The use of medicinal herbs should be standardized since their direct use could sometimes lead to severe poisoning, allergic reactions, bleeding, and cases of death. Herbal remedies may interfere with the absorption of certain necessary nutrients. Other types also increase or decrease the effect of a particular drug along with exhibiting serious side effects.^28^

**Table 1:** Plants and their major derivative compounds with anti-COX effects^21,27^

**Plant**
Ginger
Aloe vera
Epigallocatechin-3 gallate/green tea
Resveratrol
Liquorice
Milk thistle
Antioxidants present in plants (vitamins A, C, E, Se, Zn: carotenoids, flavonoids)
Boswellia
Bromelain
Garlic
Chinese skullcap
Bilberry
Grape seed extract proanthocyanidins
Panax ginseng
Curcumin

**Table 2:** Plants and their derivatives with specifically VEGF inhibitory effects^27^

**Plant**	**Plant Derivative**
Magnolia seed cones	Contains 90% honokiol
*Taxus brevifolia* (pacific yew )	Contains Taxol
*Polygonum cuspidatum* (Japanese knotweed)	Contains 20% resveratrol
*Vsicum album* (European mistletoe)	Contains mistletoe lectin III (ML3A)
*Artemisia annua* (Chinese worm wood)	Contains 95% artemisinin, and other related terpenes and flavonoids
*Curcuma longa* (turmeric)	Contains 95% curcumin
*Camellia sinensis* (green tea)	Contains 95 % phenols; 50% epigallocatechin
*Vitis vinifera* (grape seed extract)	Contains 95% proanthocyanidins
*Scutellaria baicalensis* (Chinese skullcap)	Contains 95% baicalin and flavonoids
*Silybum marianum* (milk thistle)	Contains 80% silymarin (silybin)
*Angelica sinensis* (dong quai)	Contains 4-hydroxyderricin

### Allium ascalonicum

**Figure 1:**
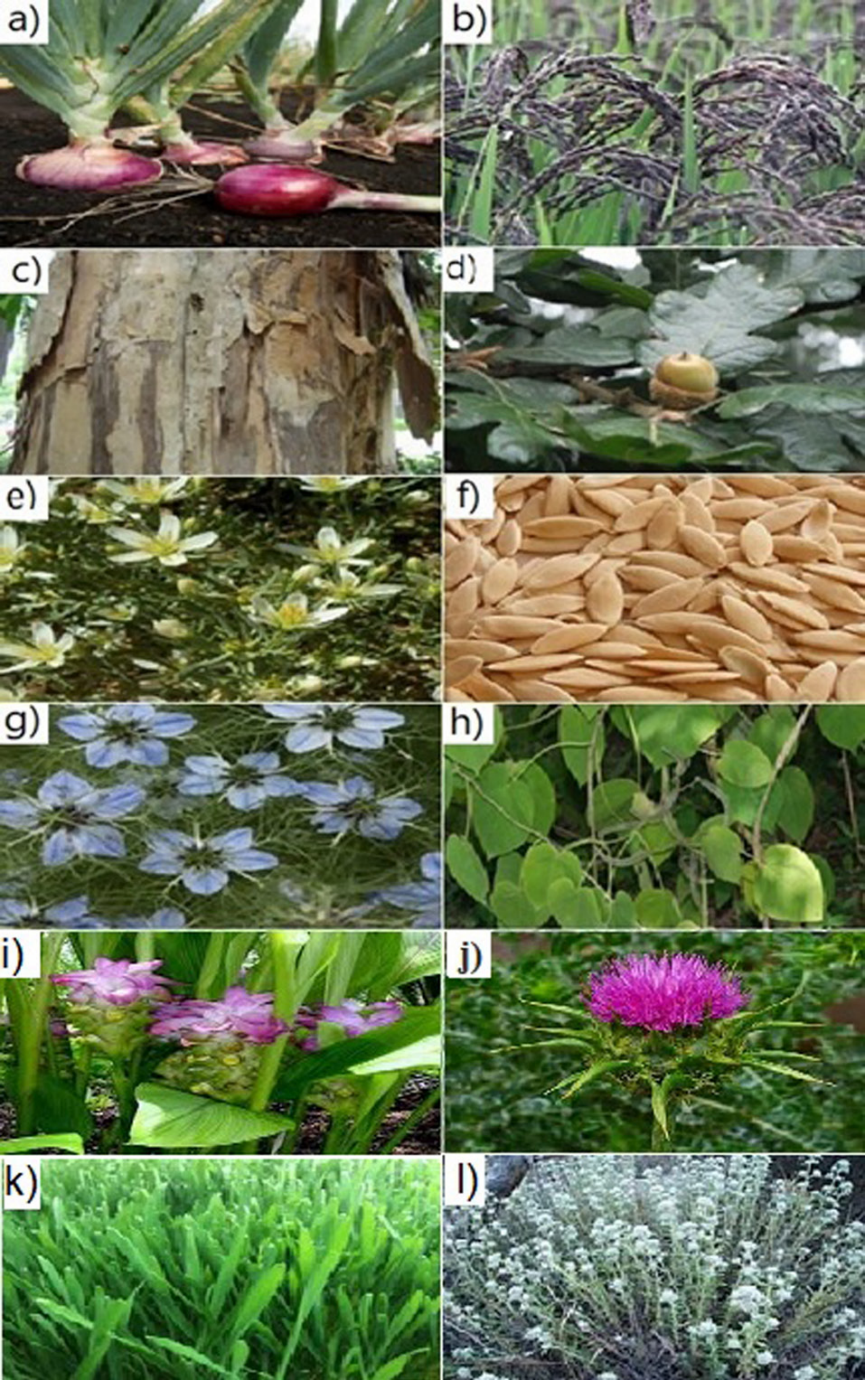
**a)** Allium ascalonicum**, b)** Black rice**, c)** Cinnamon**, d)**Oak**, e)***Peganum harmala*, **f)***Cucumis melo* seeds, **g)***Nigella sativa***, h)***Marsdenia tenacissina*, **i )***Curcuma longa***, j)***Silybum marianum*. **k)** Wheatgrass**, l)***Teucrium polium .*

*Allium ascalonicum* (Figure 1a) is considered an important species of the genus *Allium* which has long been used medicinally in many countries, including Iran. *Allium* is also used in the traditional foods. The plant has been known to retain properties like being effective on hematological indices; anti-oxidant, anti-fungal, and anti-bacterial potentials. In addition, a study on its chemical composition shows that it contains compounds such as organosulphons and polyphenols. Based on the results of the studies performed in Medical Biology Research Center, Kermanshah, Iran, on anti-angiogenic properties of Allium, it was found that the shallot rhizome extract has a significant inhibitory effect on angiogenesis. These useful features of *Allium* plant reveal its importance more than ever. Thus, given that *Allium* is routinely consumed in different communities and regarding its inhibitory effect on angiogenesis, it could be among the most convincing plant candidates for consideration in cancer treatment.^29-31^

### Black rice

Black rice (Figure 1b) contains a high level of anthocyanin and is widely used as a health-promoting food in some parts of the world. Studies have shown that black rice extract has beneficial effects against breast cancer in laboratory conditions. Anthocyanin-rich extract from black rice (AEBR) increases cytochrome C secretion which induces cell apoptosis, reduces the stability of cancer cells and also has cytotoxic effects. AEBR reduces matrix metalloproteinase 2 (MMP2), matrix metalloproteinase 9 (MMP9), and urokinase plasminogen activator (uPA) expression in mouse tumor tissues and also restrains VEGF activity and thus angiogenesis in the tumor tissue.^32-34^

### Cinnamon 

Cinnamon (Figure 1c) is a spice obtained from the inner bark of trees called *Cinnamomum*. It is used in foods as well as medicine. *Procyanidin oligomers*, as the active ingredients of cinnamon, inhibit kinase activity of purified VEGFR2. CE reduces the proliferation of cancer cells by increasing the expression of tumor necrosis factor and interferon gamma and reducing the expression of HIF which is involved in angiogenesis. EC significantly prevents transcription and translation of growth factors (EFG, VEGF, TGF-β).^35^ In a study by Kwon et al in 2010 on human melanoma cells and mouse melanoma, it was shown that cinnamon extract down-regulated activator protein 1(AP1) and nuclear factor kappa B(NF-KB) levels and also increased apoptosis rate in various cancer cells such as lymphoma, cervical cancer, and colorectal cancer.

Oral administration of cinnamon extract in a melanoma model, exerted a significant anti-tumor effect and inhibited tumor growth.^36-39^ According to available information, the plant and its derivatives have antiseptic activity and play a role in regulating apoptosis.

### Oak

Oak (Figure 1d) is a shrub from the family of beech. Oak is a plant extensively used in pharmacy. The hydro-alcoholic extract from oak’s corm shell possesses antibacterial properties. In addition, this extract reduces MMP9 expression and also inhibits VEGF secretion from tumor cells.^40^ Therefore, oak can exert inhibitory effects on tumor growth by inhibiting factors involved in angiogenesis.

### Peganum harmala

Peganum harmala (Figure 1e) is a plant of the family Nitrariaceae usually used in traditional Iranian medicine as a treatment for various types of cancers. It also has antiviral, anti-microbial, anti-nociceptive, and anti-inflammatory activities. The hydroalcoholic extract of this plant has strong anti-angiogenic effects as well; achieved through inhibiting VEGF secretion. Harmane is a naturally occurring ß-carboline extracted from Peganum harmala that can significantly decrease the expression of pro-inflammatory cytokines and pro-angiogenic factors such as NO and VEGF. The *P. harmala* extract can induce apoptosis and inhibit tumor growth in vitro by affecting BCL-2 and P-Akt genes expression. Moreover, harmane has been shown to decrease NF-KB, MMP2, and MMP9 expression. These results show that HM acts as an anti-angiogenic factor in preventing cancer.^41-43^

### Cucumis melo seeds

Melon (Figure 1f) is a native Iranian plant with cytotoxic, antioxidant, anti-inflammatory, and anti-fungal effects. Trypsin inhibitors from *C. melo* seeds (TICMS) inhibit endothelial cell migration and cell proliferation of human umbilical vein endothelial cells (HUVECs). TICMS affect the secretion of MMP2, MMP9 and VEGF from HUVEC and prevents their function. Therefore, it could be considered as an angiogenesis inhibitor.^44,45^

### Nigella sativa

*Nigella sativa* (black caraway) (Figure 1g) is an annual flowering plant in the family Ranunculaceae. It is known for its antioxidant, anti-inflammatory,^46^ immunomodulatory,^47^ and neuroprotective^48^ properties. Thymoquinone is a phytochemical compound found in *Nigella sativa* capable of inhibiting NF-KB activation and also the expression of MMPs, VEGF, and cyclin D1.

Other studies have also shown that this plant prevents transcription of the angiogenesis factors of VEGF and HIF1α. In addition, it decreases the activity level of the enzymes MMP2 and MMP9.^49,50^

### Marsdenia tenacissima

The stem of *Marsdenia tenacissima* (Figure 1h), also known as ‘Tong-guan-teng’ in traditional Chinese medicine (TCM),^51^ is often used to treat cough, expectorant, asthma, esophageal cancer, lung cancer, gastric cancer, and hepatocellular carcinoma.^52,53^ Laboratory studies indicate that the compounds found in this plant inhibit angiogenesis by reducing VEGF and MMP2,9 expressions. Moreover, it induces apoptosis in cancer cells. The use of this plant on A20 mouse lymphoma shows that *Marsdenia tenacissima* extract (MTE) associates with suppressed tumor growth and decreased angiogenesis in A20 mouse lymphoma model.^53,54^

### Curcuma longa

Curcumin is a compound extracted from the *Curcuma longa* (Figure 1i) that interacts with cancer cells in different levels. Its anti-metastatic effects are partly due to decreased MMP expression and increased TIMP1 expression. Studies have also shown that this compound inhibits the transcription of angiogenic factors of VEGF and bFGF and, in addition, inhibits NO production (in endothelial cells, which plays an important role in tumor angiogenesis and growth).^55,56^

Other activities of this combination include binding to CD13 antibody expressed by components of blood vessels and inhibiting its activity, down expression of VEGF genes, 9-MMP, and inhibition of VEGF and EGF receptors. It is also counteract the intracellular signaling pathway of tyrosine kinases.^57^

### Silybum marianum

Silymarin are polyphenolic flavonoids isolated from fruits and seeds of *Silybum marianum* (Figure 1j). Researchers have concluded that silymarin has antitumor activity by reducing VEGF and EGFR expression.

Silymarin inhibits angiogenesis and metastasis due to the accumulation of phenols via PI3. These results suggest that silymarin may be a candidate for cancer prevention.^58,59^

### Wheatgrass

Wheatgrass (Figure 1k) is a young tender grass of common wheat (*Triticum aestivum* ). Its anti-metastasis effects is partly mediated through decreasing expression of the enzymes MMP2,9 and COX-2 and also increasing the enzyme tissue inhibitor of metalloproteinases 1 (TIMP1). Studies have also demonstrated that this compound prevents the transcription of the angiogenesis factor VEGF.^60^ Wheatgrass inhibits the process of angiogenesis through accumulation of polyphenols via PI3K/AKT pathway. Thus, it could help to restrain angiogenesis and metastasis.^61^

### Teucrium polium

The *Teucrium polium* (Figure 1l) is a wild-growing flowering plant found in Europe and southwestern Asia. *T. polium* has been applied in Iranian traditional medicine for treating multitude of diseases due to its pharmacological properties. This plant has been reported to have hypolipidemic^62^ hypoglycaemic ^63^ anti-nociceptive^64^ anti-oxidant, anti-bacterial, anti-fungal, anti-septic, and anti-inflammatory^65^ potentials. There has also been an anti-angiogenic feature of *T. polium* extract reported which is attributed to a decreased NO secretion by HUVECs. Aside from blocking NO secretion and proliferation inhibition, *T. polium* can also trigger apoptosis by increasing Bax (a pro-apoptotic factor) expression and decreasing Bcl-2 expression (an anti-apoptotic factor).^63^ In 2019, Askari et al reported that the *T. polium* extract inhibited HUVEC cell growth in vitro and also VEGF secretion. These results show that *T. polium* could be a candidate for angiogenesis prevention.^66^

### Plants rich of quercetin

Quercetin is a powerful flavonoid that has a wide range of benefits to human health including its ability to reduce inflammation, relieving pain, protection against cardiovascular diseases, preventing certain cancers, and boosting the immune system. Perhaps the most important of all could be its potent antioxidant activity. This compound, like other flavonoids, binds free radicals in the body and neutralizes them before they can cause any damages. Most importantly, it inhibits angiogenesis by interacting with VEGF, cyclooxygenase 2, and lipoxygenase 5. It could also induce cancer cell death through inhibiting Akt, mTOR, and HIF-1. A study on the other hand, shows that the antioxidant property of quercetin protects endothelial cells against high glucose levels which activates an autophagy response in them. However, the precise acting mechanism of quercetin has not been fully elucidated.^67^

### Plants rich of carvacrol

Carvacrol is a phenol, a derivative of natural monoterpene with antioxidant and anticancer effects found in many plants such as wild bergamot, thyme, and tomato. Carvacrol has a potent anti-inflammatory effect exerted through inhibiting COX-2 enzyme and plays a role in reducing oxidative stress.^68^ This compound, by activating apoptosis, has an anti-proliferative effect on lung and breast cancer cells. Carvacrol significantly inhibits cell migration by blocking the phosphorylation of FAK and MMP-9 and MMP-2 and inhibited angiogenesis. In contrast, other studies have reported results of a VEGF-related angiogenesis induction by this compound. The reason behind such inconsistency could be related to the dose of carvacrol used as it reduces and inhibits MMP2 and 9 levels at high doses while increasing migration and stimulating angiogenesis at low doses.^69^

### Extracts of Chinese herbs

So far, numerous herbs that have been traditionally administered as anti-cancer medications in China are screened in various laboratory model settings to find whether they have anti-angiogenesis properties. Table 3 includes a list of plants whose anti-angiogenesis effects have been shown using bovine aortic endothelial cell culture assay as well as chicken chorioallantoic membrane model of angiogenesis.^12,70^

**Table 3:** Chinese herbs with anti-angiogenesis effects^12,58^

**Name**	**Part used**	**% Inhibition BAECC**	**% Inhibition CAM**
*Taxus chinensis*	Bark	26	-
*Catharanthus roseus*	Leaf	30	27
*Scrophularia ningpoensis*	Root	34	20
*Polygonum cuspidatum*	Whole plant	28	-
*Coptis chinensis*	Rhizome	37	25
*Berberis paraspecta*	Root	38	25
*Scutellaria baicalensis*	Root	41	2

CAM: chick embryo chorioallantoic membrane assay; BAECC: bovine aortic endothelial cell culture assay.

### Molecular mechanisms of angiogenesis

Angiogenesis process is initiated by the activation of growth factors such as VEGF, Platelet-derived growth factor, bFGF, transforming growth factor-β (TGF-β), keratinocyte growth factor, hepatocyte growth factor, ephrin-B2, and angiopoietin. Hypoxia and the activated signaling pathway of HIF in tumor cells is an important stimulator for angiogenesis. HIF-1α and HIF-2α regulate the expression of pro-angiogenic genes including TIE-2, Ang1, Ang2, and VEGF. As already mentioned, VEGF is a target gene of HIF-1α which induced-expression by HIF-1α in endothelium leads to the activation of a VEGF-related autocrine signaling pathway. This cascade is involved in the survival and proliferation of endothelial cells. VEGF on the other hand, increases the permeability of blood vessels while modulating the secretion of extracellular matrix degrading enzymes. The latter in turn leads to the expansion of the vascular system. VEGF exerts its physiologic effects through binding homologues receptors of VEGFR1 and VEGEFR2 on endothelial cells to finally activate them. Upon activation, endothelial cells secret certain types of metalloproteinases that break down the basement membrane to facilitate endothelial cell’s migration. Once the extracellular matrix is degraded and being rearranged, tubulogenesis and therefore angiogenesis are triggered by angiopoietin TIE-2, a regulator of VEGF.^66,71,72^

## Conclusion

The resistance of cancers to common treatments have always been a troublesome matter for specialists. Thus, researchers have focused a major part of their efforts on discovering and identifying new anti-cancer agents that could increase the sensitivity of cancer cells to drugs. Resistance of cancer cells to chemical medications has led to a reduction in their response to medications and as a result, failure of therapeutics. Therefore, investigating and development of more effective medications or those with fewer and less intense side-effects are of great necessity nowadays. So far, several chemical medications have been directly or indirectly derived from natural compounds found in extracts of plants. Some of the cytotoxic chemotherapy agents that are currently being administered have been designed to inhibit angiogenesis and leave the minimum toxicity in low doses. This strategy may provide a chance for patients with advanced cancers to have a longer life of a higher quality. This kind of treatment with low doses is termed metronomic dose. Metronomic model of traditional chemotherapy shows that it is also possible to administer herbal compounds reacting with the angiogenesis process to likely reinforce the positive effects of conventional chemotherapy. In other words, targeting the endothelium of vessels using non-toxic therapeutic agents with low and continuous doses can control the propagation of tumor without causing extra toxicity. The potential role of such treatments in order to extend patients’ survival and improve their quality of life needs further thorough investigation and research in clinical trials. Thus, clinical experts are also interested in identifying compounds that specifically fight different stages of angiogenesis when administered in low doses. These agents may somehow have lower toxicity in lower doses and they will most probably result in better therapeutic outcomes. The number of researches on herbs as treatments for various cancers is extensively growing because of their long-term therapeutic effects. Furthermore, researches on new herbs with anti-cancer effects look quite promising and will hopefully lead to the discovery of novel anti-cancer medications with herbal origin in the near future which would be a significant achievement in the field.

**Figure 2:**
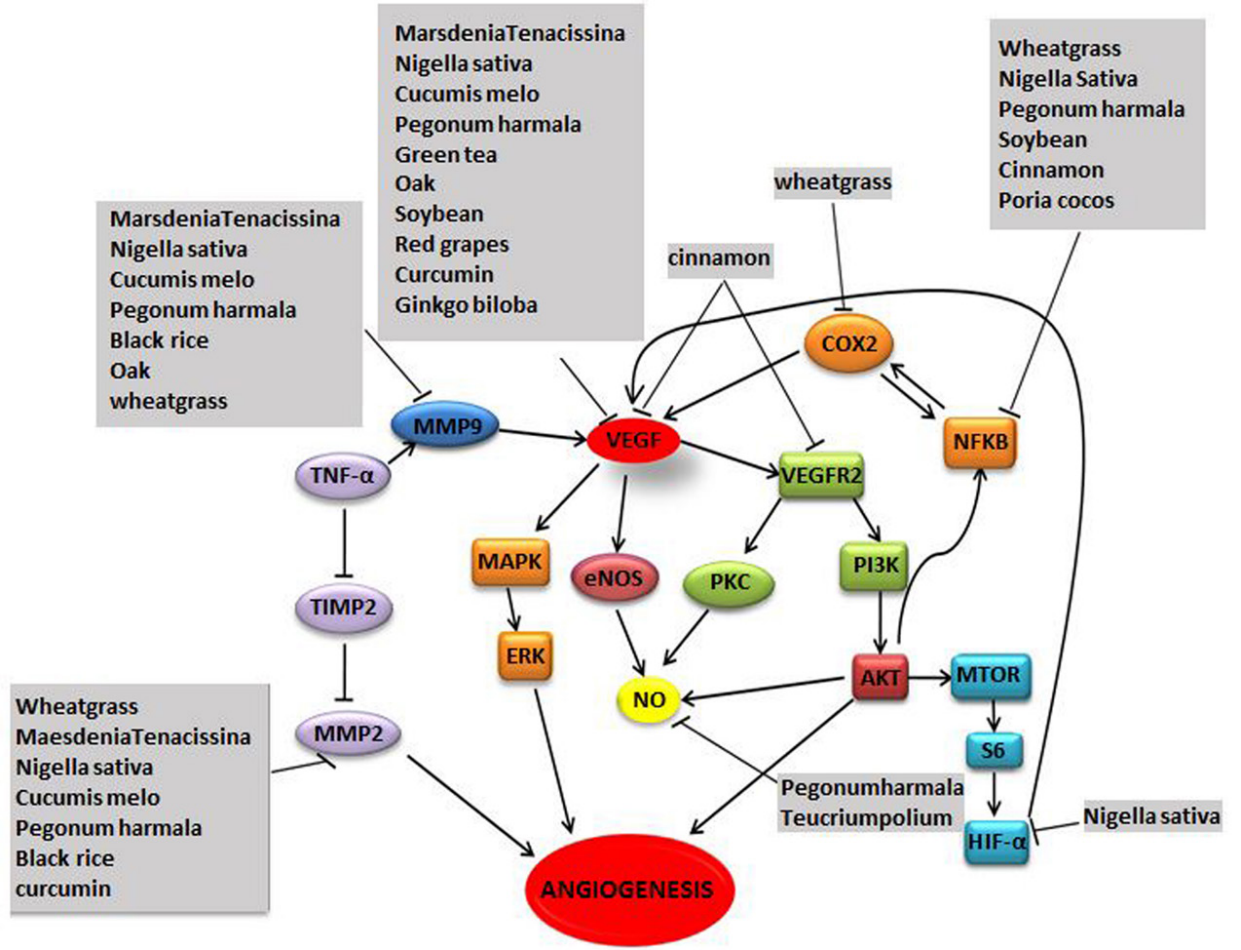
Angiogenesis pathways targeted by plants.

Several plants have been reported to exhibit anti-angiogenic properties through various molecular pathways (Figure 2 and Table 4).

**Table 4:** Angiogenesis inhibitory plants

**Plant**	**In Vitro**	**In Vivo**	**Possible mechanism**
Black rice	MCF-7, MDA-MB-231, MDA-MB-453	Xenografted MDA-MB-453 Cells in Athymic Mic	Suppresses MMP2, MMP9 and uPA expression ^31^
Cinnamon	Lymphoma, Melanoma, Cervix cancer, colorectal cancer and HUVEC	Mouse melanoma model	Suppresses VEGF and VEGFR2 expression, inhibits receptor tyrosine kinase, inhibits activation of NF-kB and AP1 signalling pathways ^34,36^
Oak	Endothelial cells	Unknown	Decreases VEGF secretion from tumor cells, Inhibits MMP9 expression in tumor cells ^38^
*Pegonum harmala*	Endothelial cells, MDA-MB-231	Unknown	Suppresses VEGF and MMP2,9 expression, inhibits activation of NF-kB^40^
*Cucumis melo* seeds	Endothelial cells	Wistar rats with gastric ulcer^73^	Inhibits VEGF and MMP2, 9 expression^42^
*Nigella sativa*	Unknown	Spinal cord injury in rats, Wistar with thickness burn rat with wound	Decreases HIF1α and VEGF expression, inhibits activation of NF-kB, decreases enzymatic activity of MMP2, 9^47,48^
*Marsdenia tenacissima*	Huvecs	Chick embryo chorioallantoic membrane (CAM)	Inhibits VEGF and MMP2, 9 expression, induces apoptosis in cancer cells^51^
Wheatgrass	HEp-2 cell	Unknown	Inhibits COX2 and MMP2, 9^53^
Teucrium polium	Huvecs	Visceral pain model in mice	Causes EC apoptosis by reducing Bcl-2 expression, reduces NO secretion by HUVECs^55^
*Poria cocos*	RAW 264.7 cells	Unknown	Inhibits activation of NF-kB^74^
Green tea	MDA-MB231, MCF7, HUVEC	C57Bl6 mice	Abrogates VEGF signaling by interfering with VEGF formation^75,76^
*Silybum marianum*	MCF-7 and MDA-MB-468, HUVEC	Ovarian cancer xenografts	Decreases EGFR and VEGF expression^58^
*Curcuma longa*	Endothelial cells macrophage	HepG2 xenografts, mouse corneal	Inhibits the transcription of angiogenic factors VEGF, EGFR and BFGF, decreased MMP expression and increased TIMP^55^

## Ethical Issues

Not applicable.

## Conflict of Interest

None declared.

## Acknowledgments

We specially thank Dr Hamid Rza Mohammadimotlagh, Rezvan Asgari and Azadeh mahnam for their valuable discussions and help with the manuscript preparation.
